# Concept mapping: Engaging stakeholders to identify factors that contribute to empowerment in the water and sanitation sector in West Africa

**DOI:** 10.1016/j.ssmph.2019.100490

**Published:** 2019-11-20

**Authors:** Elijah Bisung, Sarah Dickin

**Affiliations:** a School of Kinesiology and Health Studies, Queen's University Kingston, ON, Canada; b Stockholm Environment Institute, Stockholm, Sweden

**Keywords:** West Africa, Concept mapping, Empowerment, Gender, Water and sanitation

## Abstract

Research has shown that inadequate access to water, sanitation, and hygiene (WASH) affects women and girls in several ways, including lowering their participation in the labour market and community activities and contributing to psychosocial stress and poor educational outcomes. There is growing awareness that addressing the gender inequalities related to WASH that many women and girls face on a daily basis must go beyond focusing on delivery of infrastructure and facilities alone and include attention to issues of empowerment. Yet there is limited exploration of how the concept of empowerment is defined and applied in the WASH sector and thus limited information on how it could be measured. This study used concept mapping to uncover the meaning and key dimensions of empowerment in WASH among 34 and 24 stakeholders in Asutifi North District, Ghana, and Banfora Commune, Burkina Faso, respectively. The study was part of initial steps toward choosing indicators for developing an Empowerment in WASH Index. In Ghana and Burkina Faso, 42 and 29 items were generated, respectively. These items were thought to empower men and women in WASH at the household and community levels. In both case studies, 7 clusters were generated and named by participants, and themes related to sharing of information, sociocultural norms, participation, and accessibility of WASH services were associated with empowerment. Some themes were unique to each case study site. Participants also showed a multidimensional and multilevel understanding of empowerment. Concept mapping created an effective balance between individual and group contributions and facilitated accessible, rapid, and contextually relevant data collection. The findings can be used to generate domains of empowerment in future quantitative research as well as inform the design of the Empowerment in WASH Index.

## Highlights

• Gender empowerment is central to achieving the water-related Sustainable Development Goals.
• Participants operationalized empowerment as a multidimensional and multilevel concept.
• There were contextual similarities and differences in empowerment dimensions.
• Concept mapping provided a structured process for synthesising ideas.

## 1 Introduction

Although access to safe water and improved sanitation and hygiene (WASH) is fundamental for health and for reducing inequalities, 844 million people still lacked access to basic drinking water services and 2.3 billion people did not have access to basic sanitation services in 2015 globally (WHO/UNICEF, 2017). Biological factors and discriminatory social norms often result in women and girls bearing more costs associated with poor water and sanitation conditions (Caruso, Sevilimedu, Fung, Patkar, & Baker, 2015). In addition, these disparities may be exacerbated by intersections with other social factors such as socioeconomic status, age and disability (Hankivsky, 2012). For example, Graham, Hirai, and Kim (2016) report that among households that spend more than 30 min on water collection in 24 sub-Saharan African countries, adult women were primarily responsible for water collection in all the countries. Aside from the opportunity cost of time spent on water collection, women expend significant amounts of calories and risk contracting water-related diseases.

Considering these challenges, Sustainable Development Goal 6 (SDG 6) focuses on ensuring availability and sustainable management of water and sanitation for all people, with explicit attention to meeting the needs of women and girls. SDG 6 is critical to achieving SDG 5—“to achieve gender equality and empower all women and girls”—and has importation implications for the health and well-being of women. However, despite the importance of gender outcomes for WASH interventions, focus continues to be placed on measuring progress through improvements in WASH infrastructure, such as access to improved water sources and sanitation facilities (Lahiri-Dutt, 2015). Interventions to provide these facilities are expected to result in improved outcomes for women and girls, such as more time for productive activities and education, but such outcomes are often overlooked in monitoring. Greater understanding of the process of empowerment of women and girls in the water and sanitation sector is needed, in order to generate better evidence on the link between improved WASH services and well-being outcomes (Fisher, Cavill, & Reed, 2017; Willets et al., 2010). This study sought to develop a locally situated conceptualization of empowerment in the WASH sector in Burkina Faso and Ghana, with the view of informing the development of measurement tools.

### 1.2 Empowerment in the development sector: a brief review

Empowerment in the context of gender and development often refers to the ability of women to take control and make choices about their lives and well-being. Several definitions of empowerment are found in development discourse, with similar themes relating to expansion of agency, choices, opportunities, resources, and power (Sen, 1999b; Malhotra, 2003; Alsop & Heinsohn, 2005; Rowlands, 1997; Ibrahim & Alkire, 2007). Kabeer describes empowerment as the “processes by which those who have been denied the ability to make choices acquire such an ability” (Kabeer, 2005, p. 13). This process involves change through the use of resources to exercise human agency to achieve well-being outcomes (Kabeer, 2005). An emphasis is placed on a person's ability to define goals and to make choices to pursue them. In addition to the ability to make choices, Alsop and Heinsohn (2005) describe the institutional context or “opportunity structure” that influences how someone transforms assets into effective realization of options. In contrast, Nayraran (2005) describes empowerment from the perspective of poverty and institutions and refers to empowerment as the expansion of freedom, choices, and control over resources. This involves “expansion of assets and capabilities of poor people to participate in, negotiate with, influence, control, and hold accountable institutions that affect their lives,” (Nayaran, 2005, p. 5). Nayaran argues that the process of empowerment begins with change in institutions based on 4 key elements: access to information, inclusion and participation, accountability, and local organization capacity. The definitions and analytical lenses used in development discourse suggest that empowerment is a multidimensional and multilevel concept. Scholars have emphasized various levels and contexts of operationalizing empowerment, including at the individual level (Ewerling et al., 2017; Morgan & Coombes, 2013) household level (Alkire et al., 2013; Duvendack & Palmer-Jones, 2017) and in the context of institutions (Nayaran, 2005) and the individual, relational (household and community), and societal contexts (Huis, Hansen, Otten, & Lensink, 2017). Exhaustive reviews of empowerment definitions have been conducted by Malhotra (2003) and Ibrahim and Alkire (2007).

Over the past few decades, considerable research has emphasized the importance of empowerment for poverty reduction and development, and empowerment is often considered a major goal of development interventions and outcomes (Alkire et al., 2013; Kabeer, 2009; Nayaran, 2005). For example, food security and agriculture scholarship views women's empowerment as a critical process for improving the well-being of poor households (FAO, 2011; Garikipati, 2012; Malapit, Sraboni, Quisumbing, & Ahmed, 2015). This has led to many interventions targeting gender norms and dynamics, with the assumption that women bear a disproportionate burden of health-related outcomes associated with food insecurity, as well as a significant labour burden in many cultures, and yet do not have decision-making power over these issues and other household productive resources (Arimont et al., 2013; Garikipati, 2009; World Bank, 2012). Similarly, empowerment has been linked to utilization of maternal health services, use of modern contraception, and child nutrition outcomes (Pratley, 2016). For instance, mothers with greater financial and decision-making autonomy were found to have better infant feeding and growth outcomes than women with lower empowerment (Carlson, Kordas, & Murray-Kolb, 2015; Shroff et al., 2011).

### 1.3 Linking women's empowerment and WASH

Access to water and sanitation is thought to be critical for positive gender outcomes in low and middle-income contexts. Research has shown that inadequate access to water and sanitation affect women and girls in several ways, because of social norms that position them as principal household water collectors and managers. These effects, include low participation in the labour market and community activities, adverse biomedical outcomes, psychosocial stress, and poor educational outcomes (Bisung & Elliott, 2017; Devoto, Duflo, Dupas, Pariente, & Pons, 2012; Fink et al., 2012; Bisung & Elliott, 2014). For example, girls’ education is affected in many contexts because they skip school to collect water or to avoid managing their menstrual period in schools without adequate sanitation (Dreibelbis et al., 2013; Jasper, Le, & Bartram, 2012). In addition, the opportunity cost of water collection time is reflected in fewer hours available for childcare, leisure, and income-generating activities (Bisung & Elliott, 2014; UNICEF, 2017). When sanitation is lacking, privacy cannot be assured during menstruation or defecation. Studies have reported feelings of embarrassment, anxiety, shame, and stress due to lack of sanitation (Bisung & Elliott, 2018; Hirve et al., 2014; Hulland et al., 2015; Sahoo, Hulland, Caruso, & Swain, 2015). Despite these gender-related WASH outcomes, global commitments and many water and sanitation programs have often focused on infrastructure access rather than explicitly addressing gender differences and dynamics related to water collection labour and inequitable access to and control of water resources (Carrard, Crawford, Halcrow, Rowland, & Willetts, 2013; Seager, Robinson, van der Schaaf, Gabizon, & UN-Water Taskforce on Gender and Water, 2008).

These limitations have led to growing calls for gender mainstreaming in the water sector, with the explicit aim of addressing differing water and sanitation needs based on sex and social construction of gender,and inequalities in access to, use of, and control over water and sanitation resources at the household level and beyond (Carrard et al., 2013). However, questions remain on whether provision of or improvement in water and sanitation automatically guarantees the empowerment of women and girls. In some instances, attributing causality between an intervention and empowerment can be difficult because gender relations are embedded and mediated by other cultural and social norms (eg, ethnicity, caste, landholding status) that are difficult to account for (O’Hara & Clement, 2018). Moreover, empowerment through WASH interventions might not be sustainable without attention to other areas of daily life. For example, entrenched gender divisions in labor and gender norms related to agriculture or landholding could hinder interventions that seek to change gender dynamics related to water-collection responsibilities. There are also examples of how some water and sanitation interventions reconfigure women's daily activities in unexpected ways (O'Reilly, 2006). For example, a study conducted in Bangladesh by Sultana (2011) found that some young women took advantage of fetching water from farther places as a way to “get out of the confines of the *bari* and to socialize with others.” Water fetching thus became one of the best avenues for women to leverage outdoor mobility and participate in conversations about important things in their lives. The introduction of household tube wells, although important for improved water quality, reduced water collection time/distance and opportunities for socialization among women.

A number of frameworks, including those describing social capital (Bisung, Elliott, Schuster-Wallace, Karanja, & Abudho, 2014) and capabilities (Jepson et al., 2017), within WASH research have been used as conceptual and analytic tools. Although these frameworks provide foundation to the current study, empowerment offers a different and broader lens for water research. Indeed, capabilities and social capital frameworks invoke the centrality of human agency in addressing water issues. Empowerment offers a broader framework for understanding the synergies and interactions between agency, capabilities, and institutional opportunities (Alsop & Heinsohn, 2005; Nayaran, 2005). Narayan, for example, sees empowerment as the expansion of assets and capabilities for the poor. More importantly, empowerment draws closer to instrumentalist forms of advocacy, which is urgently needed to address current inequalities within the SDG framework. Finally, although social capital offers a unique lens for understanding how social relationships and participation influence water and sanitation outcomes and vice versa, empowerment provides conceptual clarity on how decisions regarding participation are made in the first place.

To generate better evidence on the relationship between empowerment and WASH and health outcomes, more systematic approaches are needed to track changes, such as those developed in other sectors (eg, Women's Empowerment in Agriculture Index [WEAI], a tool used extensively in monitoring agricultural initiatives under the Feed the Future Initiative) (Alkire et al., 2013). The objective of this study is to uncover the meaning and key dimensions of empowerment in WASH using local stakeholders' perspectives in 2 districts: Asutifi North in Ghana and Banfora in Burkina Faso. A better understanding of stakeholders' views on empowerment is important for developing context-specific empowerment indicators for monitoring and evaluating WASH interventions. To address the research objective, we use techniques from concept mapping, a participatory method that is useful for understanding how specific groups conceptualize specific subjects or constructs. This concept mapping approach is part of initial steps toward choosing indicators for developing an Empowerment in WASH Index. We took this participatory approach because we recognise that empowerment is context specific and normative measures of empowerment can easily reflect the values and disciplinary knowledge of the analysts instead of the contextual realities of the places and spaces in which knowledge is created (Kabeer, 1999). This concept mapping was not disaggregated by sex in order to identify general indicators of empowerment for the Empowerment in WASH Index, which will potentially be a sex-disaggregated index that can be used to identify gaps in empowerment between men and women across individual, household, and community levels.

### 1.4 Study context

Workshops were conducted in 2 sites to investigate local understanding of empowerment in Ghana and Burkina Faso. In the Asutifi North District and Banfora commune, local authorities and key WASH stakeholders launched WASH master plans in 2018 that contain a broad vision, programmes, and key strategies for implementing a 13-year initiative to achieve universal access to WASH services by 2030. This study was initiated together with IRC WASH, an international WASH nongovernmental organization (NGO) supporting the development of the district and commune master plans. Findings from this study will contribute to the development of better approaches for assessing progress toward the SDG targets for universal access to water and sanitation, with a particular focus on equity and empowerment.

Asutifi North District has a projected population of approximately 62,816 people (Ghana Statistical Service, 2014). The District is predominately rural, as 68% of the total population reside in rural areas. A majority (68%) of the residents engage in agricultural activities, although the presence of large-scale mining activities has led to a growing service industry (Ghana Statistical Service, 2014; Asutifi North District Assembly, 2018). Close to 15% of the district population do not have access to basic water services. These households mostly use water from rivers, streams, unprotected wells and springs, dugouts, and tanker services (Ghana Statistical Service, 2014). A recent survey of water infrastructure indicates that almost 42% of rural residents have access to improved (communal) water facilities. However, only 61% of such households could make a return trip of water collection within 30 min (Asutifi North District Assembly, 2018). With regard to sanitation, public toilet facilities (e.g.KVIP) are the most common, with almost half of the population relying on public facilities as their primary sanitation facility. However, close to 60% of public facilities in the district do not meet the threshold of basic sanitation (Asutifi North District Assembly, 2018), and close to 5% of the population practice open defecation in fields and bushes (Table 2).

Banfora commune, in the Cascades region of Burkina Faso, has approximately 162,000 inhabitants and comprises 22 villages and 15 urban sectors. Economic activities include agriculture, livestock husbandry, and industries such as mining, which all require reliable access to water. Surveys conducted in 2017 indicated that only 69% of the population in Banfora had access to basic water services, while 28% had access to basic sanitation services (Commune de Banfora, 2018). In the case of water services, the majority of households use boreholes with hand-operated pumps (Table 1).

**Table 1:** Water and sanitation access in Banfora and Asutifi North.

Water Ladder	% of Population Served		Sanitation Ladder	% of Population Served	
	Banfora	Asutifi North		Banfora	Asutifi North
Safely managed	0	4	Safely managed	0	10.9
Basic	69	46	Basic	28	4.7
Limited	23.3	10	Limited	4	27.3
Unimproved	0.1	40	Unimproved	60	52.5
Surface water	7.6		Open defecation	8	4.3

Sources: Commune de Banfora, 2018; Asutifi North District Assembly, 2018.

## 2 Methods

We used group concept mapping as a participatory research method to achieve the study objective. Concept mapping is built on ideas drawn from participatory planning, multivariate statistics, program theory, and theory-based judgement (Trochim, 1989; Trochim & McLinden, 2017). Trochim (1989) describes concept mapping as ‘a structured process, focused on a topic or construct of interest, entailing input from one or more participants, that produces an interpretable pictorial view of their ideas and concepts and how these are interrelated’ (Trochim & McLinden, 2017, p. 166). Participatory methods are used to create a visual display of how the participants and the group as a whole conceptualize a particular topic. Concept mapping often involves 5 main steps: 1) preparation; 2) generation of statements; 3) structuring of statements; 4) representation of statements; 5) interpretation of maps; and 6) utilization. When used with local stakeholders, the process of, and output from, concept mapping provide clues for local action and dialogue on important topics (Lebel et al., 2012). Findings driven directly by the perspectives of stakeholder groups provide researchers, and more importantly policymakers and practitioners, with potential pathways for generating appropriate locally driven interventions. As a research method, concept mapping is very structured and helps groups stay on the major tasks, improves group cohesiveness, and pictorially shows relationships between ideas that are easily interpretable by the group (O’Campo, Salmon, & Burke, 2009; Pauly et al., 2018). The concept mapping steps used in this study are presented below.

### 2.1 Preparation

The preparation often involves participant selection and other preparatory activities such as finalizing the concept mapping process, timeframe, guidelines, and instructions for generating statements, brainstorming, and structuring. A partner NGO in collaboration with the respective local government offices led the participant recruitment process. Participants were purposively selected to represent key stakeholder groups at the district level. In Ghana, participants were selected from the district assembly, queen mothers’ association, chiefs, disabled groups, women groups, youth groups, community water committees, and local government institutions such as the district departments of gender, environment, and social welfare.

In Burkina Faso, participants were selected from local government, including Banfora commune and other neighboring communes, NGOs, women's associations and other community groups. In order to maintain diversity, 2 to 4 representatives were recruited from each group. A total of 34 (14 women, 20 men) and 23 participants (8 women, 15 men) were recruited in Ghana and Burkina Faso, respectively. The study received ethics clearance from the Health Research Ethics Board at Queen's University at Kingston.

### 2.2 Generation and brainstorming

The goal of this step was to obtain a list of items related to empowerment in WASH at the household and community levels. In Ghana, stakeholders were divided into 6 groups, comprised of 5–8 members of mixed representation. To start the brainstorming exercise, participants were first asked to respond to the following focus question or prompt: *what is empowerment?* Participants were reminded that the idea is not to build consensus around the definition of empowerment but to exchange ideas on the meaning of empowerment at the household level. Responses to the focal question were written on flip charts visible to all participants. The second major activity involved brainstorming to generate factors that empower men and women in WASH at the household and community levels. Groups were encouraged to list all factors that came up during their discussions. Participants were also encouraged to respect divergent viewpoints and contributions. The list of items generated at the group level was collected by the facilitator after approximately an hour of brainstorming. Individuals were then encouraged to add any item they felt was important that was not included at the group level. This was to accommodate participants who might have been reluctant to publicly present their ideas at the group level. In Ghana, groups generated between 7 and 11 items, with a total of 63 items.

In Burkina Faso, stakeholders were divided into 4 groups made up of 5–7 members. The groups generated between 8 and 14 items, with a total of 32 items. Following the group brainstorming exercise, the research team consolidated all the group items in one final list. Duplicates were removed during the consolidation process and very similar items were grouped into one statement. Groups also examined the final list to clarify local jargons or statements that were not easily understandable to the entire group. After removal of duplicates, 42 items remained in Ghana and 29 items remained in Burkina Faso (Table 2, Table 3). Facilitation was done by 2 researchers in English and in French in Ghana and Burkina Faso, respectively. Translation into the local dialect was provided where necessary.

**Table 2:** Items and clusters generated in Banfora, Burkina Faso.

Cluster	Statement Number	Statement
Sociocultural norms		
	1	Lack of participation of women in decision making
	2	Ignorance of rights and responsibilities
	3	Sociocultural norms
	10	Influence of traditional leaders
Ownership by beneficiaries		
	4	Lack of ownership of water, sanitation and hygiene (WASH) facilities by beneficiaries
	6	Sanitation not a prioritized issue for residents
	7	Lack of engagement of the population in WASH services
Access to information, and awareness		
	5	Lack of knowledge about health risks linked to poor water and sanitation services
	13	Information, awareness of households among men and women
	28	Availability and access to information, awareness
	19	Availability of information on right to access water and sanitation
	18	Sharing of information
	26	Environmental education
Rights and inclusion of vulnerable peoples		
	8	Not taking vulnerable groups into account in the construction of water and sanitation facilities
	27	Taking into account the real needs of all users
	22	Equity and liberty in access to water and sanitation services
	25	Liberty of access
Affordability and accessibility for all		
	9	Price of water
	11	Availability of WASH services
	12	Accessibility of WASH services for everyone (people with disabilities, elderly people)
	29	Financial accessibility
Responsibility of actors in management and operation of WASH services		
	14	Management and upkeep of WASH facilities
	15	Sharing of WASH-related work
	16	Increased involvement of women (eg, training in repairing boreholes)
	17	Participation of everyone in managing WASH services
	21	Involvement of all actors in the household
	23	Participation of everyone in ensuring access and management of facilities
Efficient use of WASH resources by the household		
	20	Efficient management of WASH resources (involvement of all actors in a household)
	24	Ownership of facilities by the household to promote efficient use

**Table 3:** Items and clusters generated in Asutifi North, Ghana.

Cluster	Statement Number	Statement
Socioeconomic barriers		
	1	Lack of time related to official employment
	7	Physical disability
	11	Level of investment/income
	25	Tenancy issues
Traditions and cultural norms		
	2	Lack of time due to childcare
	3	Lack of time due to elderly care
	4	Lack of capacity during pregnancy
	15	Community norms
	16	Religious norms and beliefs
	24	Tradition
	27	Family size
	40	Gender mainstreaming
Accessibility to health and water, sanitation and hygiene (WASH) facilities		
	5	Proximity to water source
	8	Waste management
	9	Access to toilet facilities
	13	Access to menstrual hygiene/materials and change rooms for women at home and school
	17	Inequitable distribution of WASH facilities
	18	Means of transportation
	30	Accessibility to WASH services at the community level (to complete household deficits)
	32	Health/handwashing places
Education, awareness, knowledge sharing		
	6	Education
	19	Access to information
	33	Education on hygiene
	35	Access to knowledge and its sharing among the household
	38	Communication
	41	Sensitisation
Health and building plans		
	10	Health
	26	Building plans/settlement planning
Leadership and participation		
	20	Power to act
	21	Ability to take initiative
	22	Decision making
	23	Problem solving
	12	Level of motivation/laziness, doing things for themselves or others
	28	Lack of cooperation (eg, among spouses about who should invest in WASH services)
	29	Lack of cooperation at the household level
	31	Social factors/behaviour change
	34	Household access to power
	36	Household ability and willingness to manage WASH
	42	Leadership (eg, participation of women in leadership roles)
Participation and roles within households		
	14	Expectations on who is to do what
	37	Participatory decision making within households
	39	Participation/not leaving any person out

**Table 4:** Comparison of clusters by theme.

Overlap Among Clusters	Burkina Faso	Ghana
Similar		
	Sociocultural norms	Traditions and cultural norms
	Access to information, and awareness	Education, awareness, knowledge sharing
	Affordability and accessibility for all	Socioeconomic barriers
		Accessibility to health and water, sanitation, and hygiene (WASH)facilities
Less similar		
	Ownership by beneficiaries of WASH services	Participation in household roles
	Responsibility of actors in management and operation of WASH services	Leadership and participation
Different themes		
	Rights and inclusion of vulnerable peoples	Health and building plans
	Efficient use of WASH resources by the household	

### 2.3 Structuring and sorting

Each item from the final list of brainstormed items was written on a set of index cards, with the item on one side and a number on the other side. A complete set of cards was given to each group to sort into piles that made sense to them. Participants were guided by the following instructions: 1) each card can belong to only one pile; 2) all cards cannot belong to one pile; and 3) a single card cannot stand alone as a pile. This was to ensure heterogeneity across piles and avoid one-item piles. Each pile was labeled after the sorting exercise.

### 2.4 Representation

Representation involves analyzing and presenting of the statements pictorially. The statements and piles were entered into R-CMap (Bar & Mentch, 2017), a concept mapping software. We first generated a point map of all the statements. Statements that are close together on this map are likely to have been sorted together more often. Likewise, statements distant from each other were generally piled together less frequently. The point map was generated using multidimensional scaling of data from the sorted piles. The second level of analysis involved generating cluster maps to represent higher order conceptual domains (Trochim, 1989).

### 2.5 Interpretation

The interpretation focused on the cluster maps. The maps were projected on a screen and each group was asked to discuss the statements under each cluster and then suggest a name or phrase to describe the cluster. After the group naming, all the participants brainstormed together to achieve a common name for each cluster. Hybrid names were used in cases in which the participants could not agree on a single name. Each group then gave a presentation on how each domain (cluster) can serve to empower or disempower individuals at the household and community levels.

### 2.6 Rating

The last task was to allow participants to rate the statements on 2 dimensions: importance and prevalence. Each participant received the final list of statements and was asked to rate them on a Likert-type response scale (eg, l–5) based on importance and prevalence. A statement was rated based on how important it was to the participant or the perceived prevalence of the phenomena represented by the statement. Ratings ranged from 1 (not at all important or not at prevalent) to 5 (very important or very prevalent). The item and cluster ratings are shown in the supplementary material.

## 3 Results

**Fig. 1:**
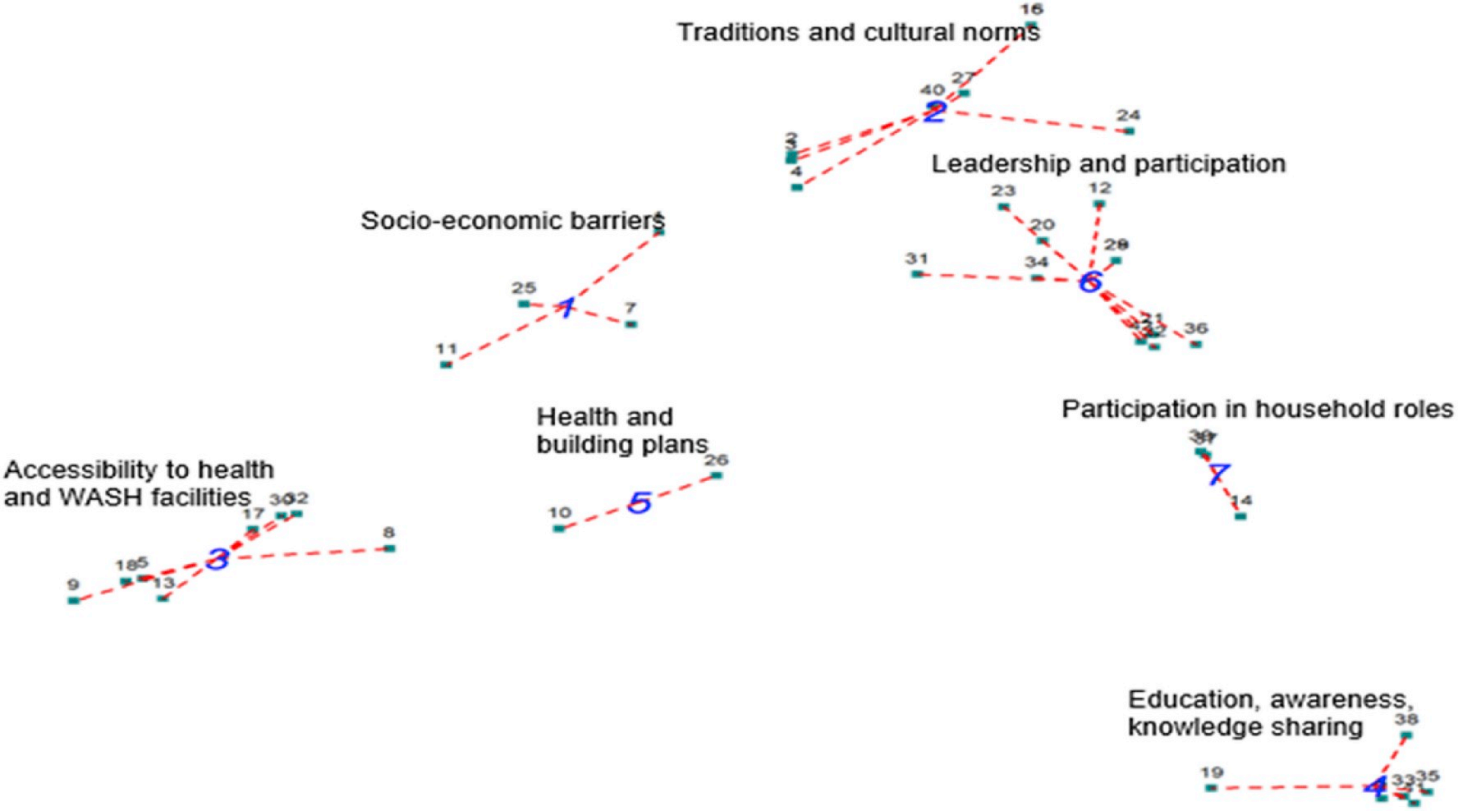
Cluster map of Empowerment in WASH dimensions in Asutifi North, Ghana.

**Fig. 2:**
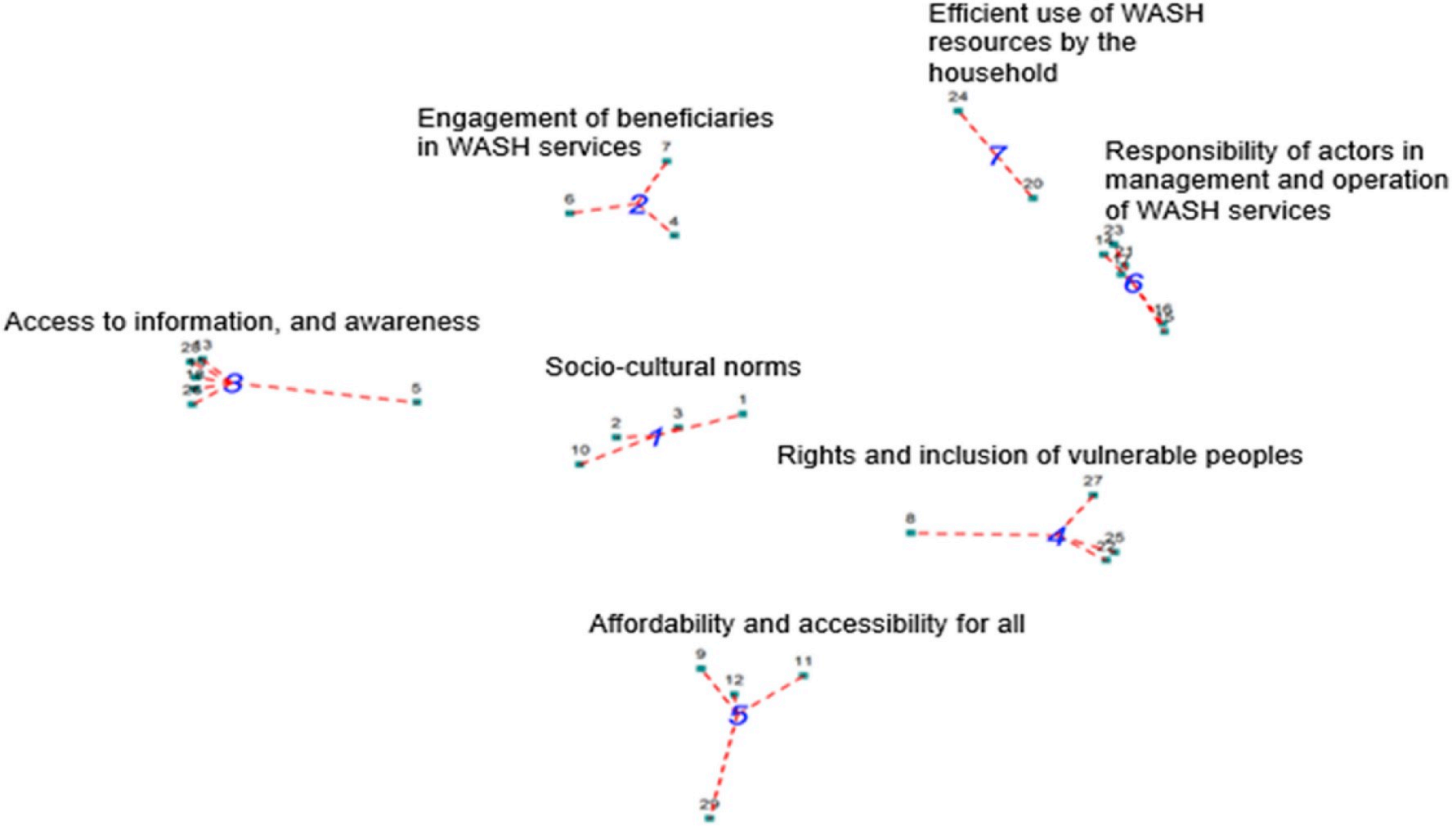
Cluster map of Empowerment in WASH in Banfora, Burkina Faso.

Final lists of 42 and 29 statements were generated by participants in Asutifi North and Banfora, respectively, after removing duplicates and led to maps of 7 clusters. Determination of the final cluster solution in both cases was driven by the subjective judgement of the research team and participants. Fig. 1, Fig. 2 show the point map and clusters generated, where each number represents a statement brainstormed by participants, and the clusters represent different conceptual spheres. Seven (7) clusters were generated in both cases, and with hierarchical cluster analysis, were used to generate the final cluster maps. Clusters that emerged in Ghana included socioeconomic barriers; traditions and cultural norms; accessibility to health and WASH facilities; education, awareness, and knowledge sharing; health and building plans; leadership and participation; and participation and roles within households. The clusters for Burkina Faso were sociocultural norms; ownership by beneficiaries; access to information and awareness; rights and inclusion of vulnerable peoples; affordability and accessibility for all; responsibility of actors in management and operation of WASH services; and efficient use of WASH resources by the household. The cluster rating maps are not shown because the clusters had very similar importance/prevalence weights.

Several similar clusters emerged in Asutifi North District and Banfora Commune (Table 4). These included sociocultural norms and traditions, access to information and awareness, affordability of WASH services, and accessibility of WASH services. Sociocultural norms, such as religious traditions and gender roles, were reported in Banfora as “old” ways of thinking and behaviours that could impede empowerment. Participants highlighted the need to “take the foot off the brake” and change mentality in order to empower people. Access to information and awareness was viewed as important for making informed choices on WASH issues, and in Asutifi North, the importance of sharing that information was emphasized. Affordability of WASH services referred to being able to afford user fees or contribute towards operations and maintenance, while accessibility was linked to the proximity and time to access WASH facilities (eg, public taps or wells) and consideration of particular needs (eg, menstrual hygiene management, age, disability).

Less similar themes were also identified. In Banfora, 2 clusters described ownership of WASH services and responsibility of all actors in management and operation of WASH. In Asutifi, rather than the concept of “*responsibilisation*,” the concept of participation was emphasized. This included 2 clusters focused on roles and participation within a household and on leadership and taking initiative. Discussion of these themes emphasized the participation of beneficiaries, including vulnerable groups, in WASH planning and programming to ensure diverse needs and interests are considered.

Additionally, several clusters were unique to the respective cases. In Asutifi North, a cluster corresponding to health services and land tenure regulations emerged, tying WASH services to the broader institutional context. In Banfora, a cluster emphasized citizen rights to WASH and another emphasized the efficient use of WASH-related resources. These clusters in Banfora emphasized the realization of rights, as well as the duties and responsibilities of WASH users.

## 4 Discussion

Some level of association is often assumed to exist between access to WASH facilities and women's empowerment. Yet studies seldom reflect on the pathways through which access leads to empowerment or vice versa. Evidence from the literature on water security has shown that the use of “access” in a generic way obscures important inequities and capacities that provide context for linking access to empowerment or empowerment to access (Aleixo et al., 2018; Coffey et al., 2014; Gimelli, Bos, & Rogers, 2018). For instance, a connection between water or sanitation facilities on premises and women's autonomy and preference to use such facilities is often made (Coffey et al., 2014). A case in Northeast Brazil reported that water collection from fecally contaminated sources continued even after a water supply system was constructed; however, where a woman was a household head and thus had greater decision-making power over choice of sources, there was a higher tendency to use higher quality sources (Aleixo et al., 2018). Further, research from India and Bangladesh has shown that newly married women intrinsically value water collection because it provides an opportunity to leave the confines of the home and to socialize (Narain, 2014; Sultana, 2011). Although the health benefits from reduced water collection time/burden may outweigh those gained from socialization, such examples, as argued by Kabeer (1999), require that future indicators of empowerment based on “access” must be defined in ways that clearly show the potential for strengthening human agency, power, and value achievement.

Group concept mapping has been applied in a range of health contexts, such as patient-centred care and evaluation (Bayer, Cabrera, Gilman, Hindin, & Tsui, 2010; Ogden, Barr, & Greenfield, 2017; Stoyanov et al., 2012). In this study, the methodology provided a structured approach to conceptualize the complex concept of empowerment in the WASH sector among local stakeholders, a concept that has received little thorough exploration despite the emphasis on gender outcomes in WASH interventions. We conducted case studies in Banfora Commune, Burkina Faso, and Asutifi North District, Ghana, where participants reported a range of factors that contribute to empowerment in WASH. The clusters identified in both case studies reinforce the idea that empowerment is a multidimensional concept that can be operationalized at multiple levels (Huis et al., 2017). Despite our original focus on household- and community-level WASH issues, participants also engaged with issues related to the broader institutional context, particularly the role of WASH service providers. These 3 levels are used to interpret the clusters.

Within the household and community, the findings highlight differing conceptualizations of agency, inclusion, and empowerment based on the cultural context, with a greater emphasis on rights and responsibility in Banfora Commune and on participation in Asutifi North District. For instance, at the household level, participants in Asutifi North District emphasized participation and inclusion of all household members in decision making. In Banfora, there was an emphasis on ownership and the responsibilities of all actors to play their parts in management of WASH. This included sharing of work at the household level and carrying out respective responsibilities, while at the community level it involved greater participation in WASH responsibilities, such as greater engagement of women in WASH management. In Asutifi North, the concept of participation at the community level was related to leadership and taking initiative on decisions, as well as a household's access to power. These differences may be linked to the French translation of empowerment to *autonomisation* that was used in the Banfora concept mapping workshop emphasizing autonomy, compared with the concept of access to power that emerged in the Asutifi North workshop conducted in English. Thus, while these factors can lead to (dis)empowerment and influence the outcomes of WASH interventions, how they are manifested and measured might differ in the 2 contexts.

The broader social, cultural, and economic contexts also emerged as something that could constrain empowerment and agency to make WASH-related decisions or realize rights. For instance, in Asutifi North District, socioeconomic factors were reported, such as low income, physical disability, time use, and tenancy regulations. These factors have wider impacts on how people access, negotiate, and use WASH services. Although the clusters related to these issues seem outside the domain of the household, they influence women's empowerment in complex ways. For example, prevailing human rights, local governance systems, and economic circumstances affect women's decision-making power or participation in WASH activities. Thus, empowering men and women in WASH requires close attention to interventions targeted at several levels and areas (eg, household, community, service providers). In addition, sociocultural norms and traditional behaviours and practices emerged as cluster in both cases, as these norms were viewed constrains to empowerment, such as limiting opportunities for women in decision making at the household and community levels. It is important to note that some of the clusters presented here have been identified as domains of empowerment in the broader literature (Goodman, Elliott, Gitari, Keiser, & Raimer-Goodman, 2016; Durgaprasad and Sivaram, 2007; Kema, Komwihangiro, & Kimaro, 2012; Hassan et al., 2012; van Welie & Romijn, 2018). For example, past studies have identified access to information, participation, community engagement, and improved knowledge as important domains of empowerment (Goodman et al., 2016; van Welie & Romijn, 2018; Hassan et al., 2012). We used these dimensions and the results of this study to inform the development of indicators for piloting an Empowerment in WASH Index (EWI)*.* Similar to the Women Empowerment in Agriculture Index *(*WAEI), the EWI will be a multidimensional instrument that can be used to measure an individual's empowerment in WASH at several levels and across several domains.

### 4.1 Towards a conceptual framework of empowerment in WASH

Existing frameworks for understanding empowerment often utilize a multilevel approach (Huis et al., 2017) and offer different roadmaps for how to operationalize and measure empowerment. However, limited theoretical conceptualizations of the process of empowerment within a WASH context have been developed. Existing approaches or frameworks are oriented towards assisting practitioners with gender mainstreaming in WASH interventions. For instance, Carrard et al. (2013) describe spaces where gender equality outcomes may be situated, characterized by 2 axes of change, individual changes or relational changes, and changes occurring in private or public spaces.

**Fig. 3:**
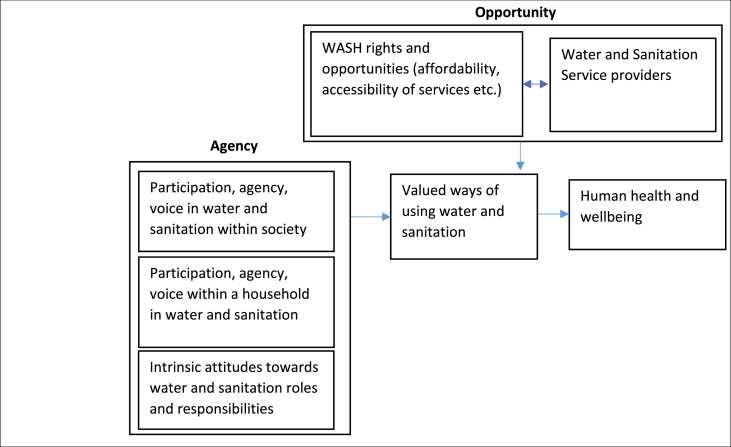
Conceptual framework of empowerment in water, sanitation, and hygiene (WASH).

We draw on the results of the concept mapping study and on existing theoretical constructs describing empowerment to generate a conceptual framework of empowerment in WASH (Fig. 3). We describe a multilevel conceptualization as supported by the case study results but also indicating possible interlinkages of dimensions. The framework describes a process of empowerment that centres on an interaction between agency and opportunity, drawing on the work of Kabeer (2005) and Alsop and Heinsohn (2005). Opportunity describes an individual's rights and opportunities to secure WASH, which interacts closely with WASH services provided by local authorities and other service providers. Agency of users can occur at the individual level (eg, intrinsic attitudes and behaviors), at the household level (eg, sharing of work, responsibilities), and at the community level (eg, participation in managing WASH or in interacting with service providers). Together, participation and agency and the achievement of rights and opportunities mean that an individual is empowered to use water and sanitation in ways that they value, going beyond simplistic descriptions of access. This process of empowerment in turn contributes to human health and well-being, as demonstrated by other frameworks that link ability to conduct valued functionings in relation to water and sanitation to human well-being (Jepson et al., 2017; Mehta, 2014). The framework recognizes that agency and opportunities can be constrained by sociocultural norms and other barriers. In particular, gender norms often constrain rights to safe water and sanitation, such as accessibility, adequate quantity, or affordability.

### 4.2 Limitations

This study has some limitations worth acknowledging. Given that empowerment is a complex concept and is less conceptualized in the WASH sector, there were some conceptual challenges in exploring its dimensions through concept mapping. For example, comparison between the study sites was challenging because some of the clusters emerged based on experiences grounded in the local environment. Thus, the clusters must be interpreted in the light of each district's specific context. The difference in the cluster maps suggests a need for caution in assuming that empowerment or dimensions of empowerment can be uniformly defined across contexts or cultures. In addition, the prompt question used in the workshop focused on household and community levels, where many WASH decisions and participation by users take place, but it did not explicitly explore individual conceptualization of empowerment or the broader institutional sphere.

Although the concept mapping approach is suitable for capturing and representing the views of a broad group of stakeholders, the results are dependent on the type of group that participates. The study involved adult participants largely drawn from various stakeholder groups in the study sites. It would be important to consider participants outside this category who face unique WASH challenges. In addition, we are unable to present sex disaggregated data and cluster maps because we did not conduct gender-specific group concept mapping. Finally, balancing the time required for concept mapping discussions with the time demands of the various stakeholders was a practical challenge. Further discussions could have led to modification of clusters or in-depth discussions of pathways linking each statement/cluster with empowerment.

## 5 Conclusions

Substantial research has focused on the importance of women's empowerment for poverty reduction and development but overlooked how it occurs in the WASH sector. There are strong potential synergies between SDG 6 and SDG 5, as achieving universal access to safe water and sanitation services (6.1 and 6.2) in households, healthcare centres, schools, and workplaces underpins several targets on gender equality and empowerment. However, provision or improvement in water and sanitation services may not automatically guarantee the empowerment of women and girls in all contexts. Likewise, reducing gender inequalities and empowering women and girls is thought to be critical for the success of WASH interventions, but less research has explored these pathways (Taukobong et al., 2016). The limited conceptualization of empowerment in the WASH sector hinders the development of tools for monitoring progress on associated gender outcomes and understanding the broader contributions of such interventions to human wellbeing. This study advances understanding of women's empowerment in the WASH sector and illustrates factors within the household, community, and institutional levels that influence empowerment. The clusters identified through concept mapping in this study can be used to inform indicators for measuring empowerment and related gender measures in the WASH sector. As countries track process toward achieving universal access to WASH services, this type of information will allow researchers and practitioners to identify and act on pathways through which empowerment contributes to achieving these targets.

## Ethics statement

The study received ethics clearance from the Health Research Ethics Board at Queen's University, Kingston.

## Funding

This work was supported by an accelerated grant provided by the REACH programme led by Oxford, which is itself funded by UK aid from the UK Department for International Development (DFID). Funding was also provided by the Swedish International Development Cooperation Agency to the Stockholm Environment Institute.

## Declaration of competing interest

Authors declare no conflict of interest.
