# Supplementary material for: Concept mapping: Engaging stakeholders to identify factors that contribute to empowerment in the water and sanitation sector in West Africa
Source: SSM Popul Health. 2019 Nov 20;9:100490. doi: 10.1016/j.ssmph.2019.100490 (PMC6978480; doi:10.1016/j.ssmph.2019.100490)
Supplement: Multimedia component 1 [file mmc1.docx]

**Supplementary data**

**Table 1: Brainstormed Items in Clusters with Importance and Prevalence Ratings, in Banfora**

| **Cluster** | **Statement Number** | **Statement** | **Prevalence** | **Prevalence by Cluster** | **Importance** | **Importance by Cluster** |
| --- | --- | --- | --- | --- | --- | --- |
| Sociocultural norms |  |  |  | 3.83 |  | 3.76 |
|  | 1 | Lack of participation of women in decision making | 3.80 |  | 4.00 |  |
|  | 2 | Ignorance of rights and responsibilities | 4.10 |  | 3.89 |  |
|  | 3 | Socio cultural norms | 3.90 |  | 4.00 |  |
|  | 10 | Influence of traditional leaders | 3.50 |  | 3.15 |  |
| Engagement of beneficiaries in water, sanitation and hygiene (WASH) services |  |  |  | 3.95 |  | 4.22 |
|  | 4 | Lack of ownership of WASH facilities by beneficiaries | 3.95 |  | 4.30 |  |
|  | 6 | Sanitation not a prioritized issue for residents | 4.15 |  | 4.30 |  |
|  | 7 | Lack of engagement of the population in WASH services | 3.74 |  | 4.05 |  |
| Access to information, and awareness |  |  |  | 3.66 |  | 4.25 |
|  | 5 | Lack of knowledge about health risks linked to poor WASH services | 4.25 |  | 4.20 |  |
|  | 13 | Information, awareness of households among men and women | 4.05 |  | 4.50 |  |
|  | 28 | Availability and access to information, awareness | 3.74 |  | 4.35 |  |
|  | 19 | Availability of information on right to access WASH | 3.42 |  | 4.20 |  |
|  | 18 | Sharing of information | 3.00 |  | 4.15 |  |
|  | 26 | Environmental education | 3.53 |  | 4.10 |  |
| Rights and inclusion of vulnerable peoples |  |  |  | 3.61 |  | 4.08 |
|  | 8 | Not taking vulnerable groups into account in the construction of WASHfacilities | 4.10 |  | 3.95 |  |
|  | 27 | Taking into account the real needs of all users | 3.53 |  | 4.15 |  |
|  | 22 | Equity and liberty in access to WASHservices | 3.44 |  | 4.26 |  |
|  | 25 | Liberty of access | 3.39 |  | 3.95 |  |
| Affordability and accessibility for all |  |  |  | 3.80 |  | 4.12 |
|  | 9 | Price of water | 4.00 |  | 3.80 |  |
|  | 11 | Availability of WASH services | 3.89 |  | 4.53 |  |
|  | 12 | Accessibility of WASH services for everyone (people with disabilities, elderly people) | 3.79 |  | 4.42 |  |
|  | 29 | Financial accessibility | 3.53 |  | 3.75 |  |
| Responsibility of actors in management and operation of WASH services |  |  |  | 3.35 |  | 4.18 |
|  | 14 | Management and upkeep of WASH facilities | 4.11 |  | 4.55 |  |
|  | 15 | Sharing of WASH-related work | 3.16 |  | 3.68 |  |
|  | 16 | Increased involvement of women (eg, training in repairing boreholes) | 2.95 |  | 3.85 |  |
|  | 17 | Participation of everyone in managing WASH services | 3.11 |  | 4.40 |  |
|  | 21 | Involvement of all actors in the household | 3.32 |  | 4.30 |  |
|  | 23 | Participation of everyone in ensuring access and management of facilities | 3.47 |  | 4.30 |  |
| Efficient use of WASH resources by the household |  |  |  | 3.40 |  | 4.33 |
|  | 20 | Efficient management of WASH resources (involvement of all actors in a household) | 3.47 |  | 4.40 |  |
|  | 24 | Ownership of facilities by the household to promote efficient use | 3.33 |  | 4.25 |  |

**Table 2: Brainstormed Items in Clusters With Importance and Prevalence Ratings, in Asutifi North**

| **Cluster** | **Statement Number** | **Statement** | **Prevalence** | **Importance** | **Cluster Mean Prevalence** | **Cluster Mean Importance** |
| --- | --- | --- | --- | --- | --- | --- |
| Socioeconomic barriers |  |  |  |  | 3.81 | 3.98 |
|  | 1 | Lack of time related to official employment | 3.69 | 3.46 |  |  |
|  | 7 | Physical disability | 4.20 | 4.37 |  |  |
|  | 11 | Level of investment/income | 4.12 | 4.46 |  |  |
|  | 25 | Tenancy issues | 3.22 | 3.63 |  |  |
| Traditions and cultural norms |  |  |  |  | 3.96 | 3.90 |
|  | 2 | Lack of time due to childcare | 3.68 | 3.52 |  |  |
|  | 3 | Lack of time due to elderly care | 3.64 | 3.44 |  |  |
|  | 4 | Lack of capacity during pregnancy | 3.81 | 3.81 |  |  |
|  | 15 | Community norms | 4.11 | 4.08 |  |  |
|  | 16 | Religious norms and beliefs | 4.15 | 4.16 |  |  |
|  | 24 | Tradition | 4.22 | 3.88 |  |  |
|  | 27 | Family size | 3.83 | 3.84 |  |  |
|  | 40 | Gender mainstreaming | 4.26 | 4.43 |  |  |
| Accessibility to health and water, sanitation and hygiene (WASH) facilities |  |  |  |  | 4.15 | 4.49 |
|  | 5 | Proximity to water source | 4.44 | 4.88 |  |  |
|  | 8 | Waste management | 4.44 | 4.69 |  |  |
|  | 9 | Access to toilet facilities | 4.24 | 4.81 |  |  |
|  | 13 | Access to menstrual hygiene/materials and change rooms for women at home and school | 3.81 | 4.11 |  |  |
|  | 17 | Inequitable distribution of WASH facilities | 4.15 | 4.44 |  |  |
|  | 18 | Means of transportation | 3.74 | 3.96 |  |  |
|  | 30 | Accessibility to WASH services at the community level (to complete household deficits) | 4.32 | 4.67 |  |  |
|  | 32 | Health/handwashing places | 4.09 | 4.35 |  |  |
| Education, awareness, knowledge sharing |  |  |  |  | 4.20 | 4.48 |
|  | 6 | Education | 4.35 | 4.78 |  |  |
|  | 19 | Access to information | 4.04 | 4.46 |  |  |
|  | 33 | Education on hygiene | 4.17 | 4.50 |  |  |
|  | 35 | Access to knowledge and its sharing among the household | 3.96 | 4.29 |  |  |
|  | 38 | Communication | 4.26 | 4.40 |  |  |
|  | 41 | Sensitisation | 4.43 | 4.43 |  |  |
| Health and building plans |  |  |  |  | 4.07 | 4.56 |
|  | 10 | Health | 4.28 | 4.73 |  |  |
|  | 26 | Building plans/settlement planning | 3.87 | 4.39 |  |  |
| Leadership and participation |  |  |  |  | 4.04 | 4.13 |
|  | 20 | Power to act | 3.96 | 3.96 |  |  |
|  | 21 | Ability to take initiative | 4.04 | 4.24 |  |  |
|  | 22 | Decision making | 4.08 | 4.46 |  |  |
|  | 23 | Problem solving | 4.29 | 3.79 |  |  |
|  | 12 | Level of motivation/laziness, doing things for themselves or others | 3.69 | 3.85 |  |  |
|  | 28 | Lack of cooperation (eg, among spouses about who should invest in WASH services) | 3.96 | 3.80 |  |  |
|  | 29 | Lack of cooperation at the household level | 4.04 | 3.92 |  |  |
|  | 31 | Social factors/behaviour change | 4.26 | 4.50 |  |  |
|  | 34 | Household access to power | 3.96 | 3.83 |  |  |
|  | 36 | Household ability and willingness to manage WASH | 3.91 | 4.68 |  |  |
|  | 42 | Leadership (eg, participation of women in leadership peoples) | 4.30 | 4.42 |  |  |
| Participation in household roles |  |  |  |  | 4.01 | 4.32 |
|  | 14 | Expectations on who is to do what | 3.81 | 4.15 |  |  |
|  | 37 | Participatory decision making within households | 4.00 | 4.29 |  |  |
|  | 39 | Participation/not leaving any person out | 4.22 | 4.52 |  |  |
